# Oxygen-Binding Sites of Enriched Gold Nanoclusters for Capturing Mitochondrial Reverse Electrons

**DOI:** 10.1021/acs.nanolett.4c02331

**Published:** 2024-08-29

**Authors:** Fang-Hsuean Liao, Shu-Ping Chen, Chun-Nien Yao, Te-Haw Wu, Meng-Ting Liu, Chia-Shuo Hsu, Hao Ming Chen, Shu-Yi Lin

**Affiliations:** † Institute of Biomedical Engineering and Nanomedicine, National Health Research Institutes, Zhunan Town 35053, Taiwan; ‡ Department of Chemistry and Center for Emerging Materials and Advanced Devices, National Taiwan University, Taipei 106319, Taiwan; § National Synchrotron Radiation Research Center, Hsinchu 300092, Taiwan; ∥ Department of Chemistry, National Tsing-Hua University, Hsinchu 300044, Taiwan

**Keywords:** Low-coordination, gold nanoclusters, oxygen intercalation, CoQ, reverse electron transfer

## Abstract

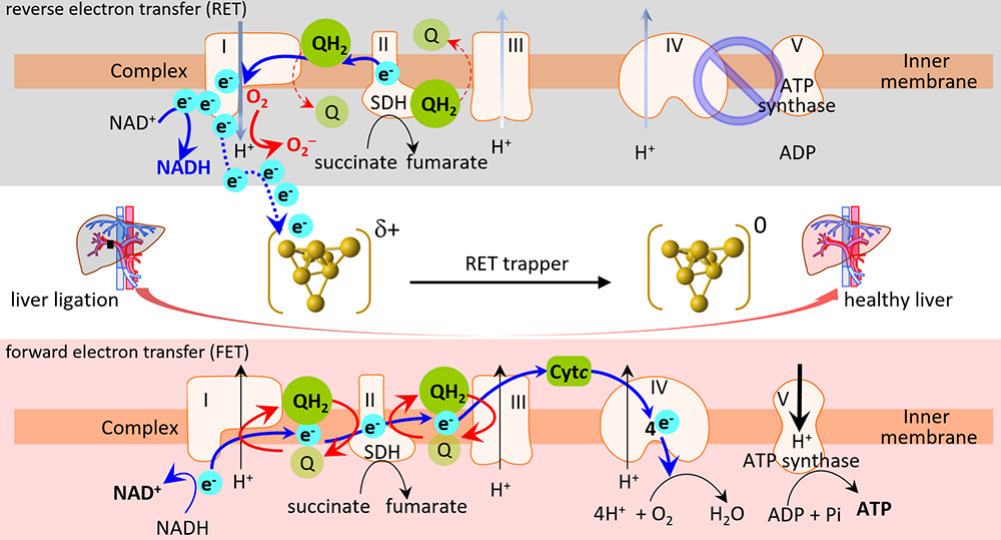

Reverse electron transfer (RET), an abnormal backward flow of electrons from complexes III/IV to II/I of mitochondria, causes the overproduction of a reduced-type CoQ to boost downstream production of mitochondrial superoxide anions that leads to ischemia-reperfusion injury (IRI) to organs. Herein, we studied low-coordinated gold nanoclusters (AuNCs) with abundant oxygen-binding sites to form an electron-demanding trapper that allowed rapid capture of electrons to compensate for the CoQ/CoQH_2_ imbalance during RET. The AuNCs were composed of only eight gold atoms that formed a Cs-symmetrical configuration with all gold atoms exposed on the edge site. The geometry and atomic configuration enhance oxygen intercalation to attain a d-band electron deficiency in frontier orbitals, forming an unusually high oxidation state for rapid mitochondrial reverse electron capture under a transient imbalance of CoQ/CoQH_2_ redox cycles. Using hepatic IRI cells/animals, we corroborated that the CoQ-like AuNCs prevent inflammation and liver damage from IRI via recovery of the mitochondrial function.

Mitochondrial reverse electron transport (RET), which boosts excessive mitochondrial superoxide anions, is often caused by the ischemia-reperfusion process following ischemic stroke, acute myocardial infarction, heart attack, or organ transplantation.^1^ RET can lead to severe secondary tissue damage and organ dysfunction.^2^ The mitochondrion, known as the energy factory of the cell, is a double membrane-bound organelle with multiple transmembrane protein complexes of which the protein complexes I/II/III/IV comprise the mitochondrial electron transport chain (ETC), also called the mitochondrial respiratory chain.^3^ Mitochondria play pivotal roles in bioenergetics and cell physiological functions.^4^ The supercomplex assembly of the ETC performs a series of redox cycles composed of several molecules, protons, electrons, ubiquinone/ubiquinol (CoQ/CoQH_2_), and O_2_ in order to synthesize the final energy product, ATP.^5^ During this process, electrons flow from respiratory complex I (also known as NADH/ubiquinone oxidoreductase) and complex II (succinate/ubiquinone oxidoreductase) as a forward electron transfer through a cascade of the CoQ/CoQH_2_ pool and the cytochrome oxidase complex to catalyze the reduction reaction of O_2_ to form the final product, water.^6^

Once the ischemia-reperfusion process occurs, the forward electron flow of mitochondria can reverse direction from complex III/IV back to complex II, CoQH_2_, and complex I. The RET process leads to a burst of intracellular superoxide anions production that causes mitochondrial dysfunction and ischemia-reperfusion injury (IRI) to organs.^7^ In addition, complex II of the ETC has multisubunit structures, which are involved in several electron transfer processes, such as succinate dehydrogenase (SDH), flavin adenine dinucleotide (FAD), and iron–sulfur clusters.^8^ Under normal conditions, SDH catalyzes the oxidation of succinate to fumarate and feeds electrons to the CoQ pool by being incorporated into FAD and complex III, thereby triggering the CoQ/CoQH_2_ redox cycle and promoting the mitochondrial respiratory chain.^6^ Conversely, RET can be accompanied by the excessive generation of mitochondrial superoxide anions, and the increased production of NADH switched from NAD^+^ in complex I bypasses the transient dysfunction of the redox cycling of CoQ/CoQH_2_. However, in IRI-induced RET, the synergetic effect of enzymatic redox cycles within complex II/III is interrupted, which results in CoQH_2_ domination of the CoQ/CoQH_2_ pool and affects mitochondrial electron supply and demand.^9^ To avoid RET-induced abnormal mitochondrial conditions, the development of a CoQ-mimicking supplement or medicine as an electron acceptor is necessary to prevent the increase in reverse electron flow backward to complex I, thereby decreasing RET-induced organ injuries.

Despite developing natural compounds, such as quinone, that are similar to CoQ10 analogues, their bioactivation is insufficient for use in the IRI treatment.^10^ Recently, low-coordination nanoparticles with enzymye-like activity have been shown to have abundant edge defects that can dope other elements to promote catalytic efficiency.^11−19^ The intercalation of oxygen on edge sites leads to the formation of an electron-demanding trapper, which might be a new strategy to design an artificial CoQ for RET capture. Herein, we found ultrasmall gold nanoclusters (AuNCs) with planar or nonplanar configuration that might offer low-coordinated unsaturated sites on the edge similar to the assembly of single atoms,^20−23^ which may possess better reactivity to be suitable as RET-trapper candidates. To do that, we used a well-established template to gather the gold atoms for the preparation of low-coordinated AuNCs.^24,25^ It should be emphasized that our previous studies listed in Table S1 only showed how to synthesize and enhance intrinsic fluorescence, intracellular tracking, and biocompatibility of AuNCs, but their geometry and atomic configuration in response to the inherent catalytic activity remained unclear. Through a deep molecular understanding, we thus further hypothesized the potential preventive effect of electron-deficient AuNCs on mitochondrial functions in the correlation between the formation of mitochondrial superoxide anions and RET for treating the IRI in upstream pathological states (Scheme 1).

**Scheme 1:**
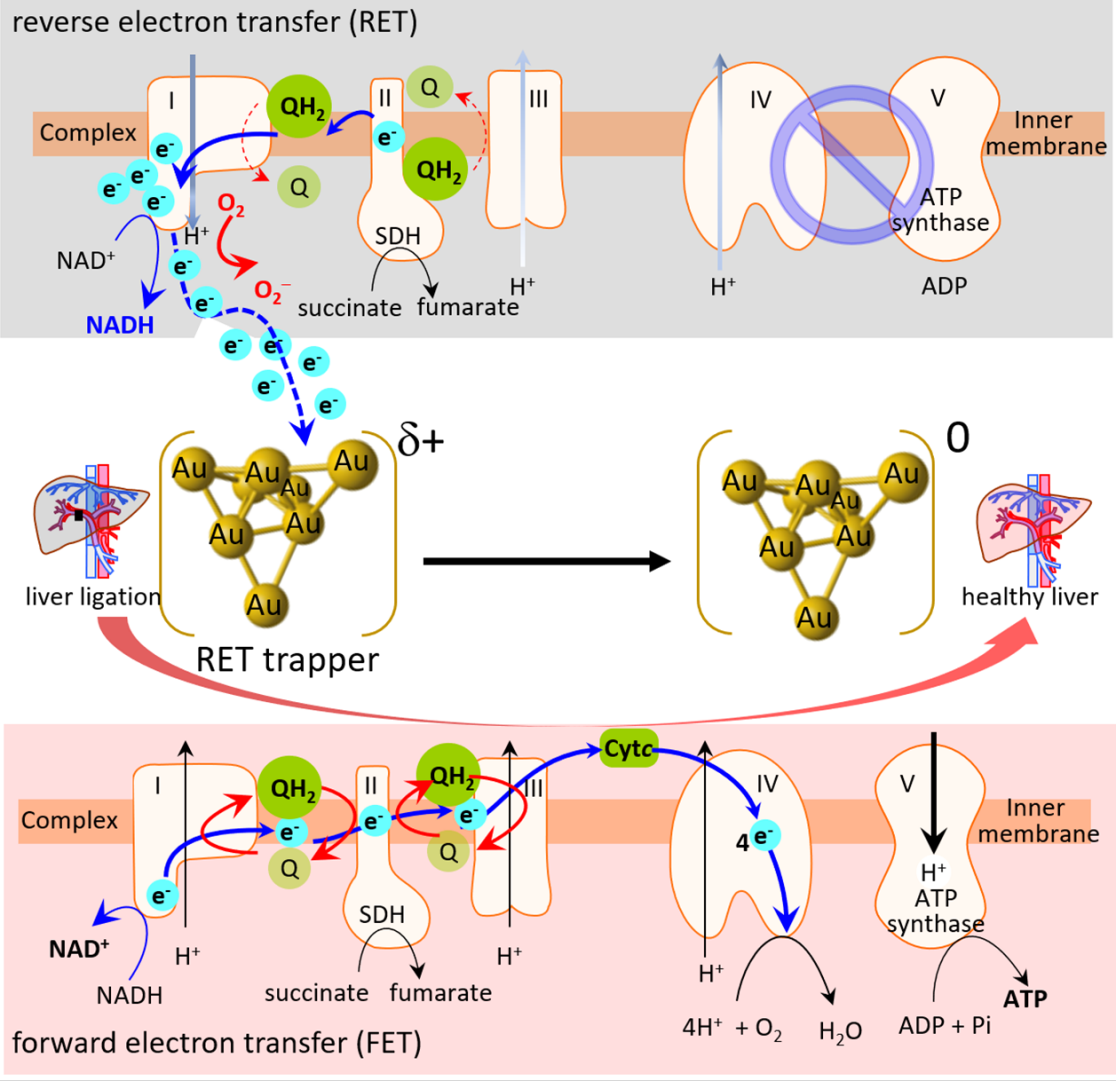
Schematic Illustration of the Mechanism of Inhibition of Reverse Electron Transfer (Gray Color) by the Electron Acceptor of Low-Coordinated Gold Nanoclusters (AuNCs) with a High-Oxidation State Forming the Au^δ+^ Cluster to Restore the Forward Electron Chain Reaction (Pink Color) and Eventually Avoid Mitochondrial Dysfunction after Ischemia-Reperfusion Injury (IRI) of the Liver QH_2_ and Q represent CoQH_2_ and CoQ, respectively. The liver illustration was recreated using illustration toolkits purchased from Motifolio.

X-ray absorption spectroscopy is an indispensable method to probe an atomic configuration without long-range order, such as that of AuNCs.^26,27^ The X-ray absorption near edge structure (XANES) spectrum displays remarkable features of electronic transitions from the core levels to unoccupied states of AuNCs, thereby reflecting the electronic structures of the frontier orbitals (e.g., oxidation state) of Au as illustrated in Figure 1a. On the basis of the formal electronic configurations of Au, which are Au^0^([Xe]4f^14^5d^10^6s^1^), Au^1+^([Xe]4f^14^5d^10^6s^0^), and Au^3+^([Xe]4f^14^5d^8^6s^0^), only Au^3+^ is expected to exhibit the 2p_3/2_ → 5d transition. However, under most conditions, the proximity of the Au 5d energy level to the 6s level can lead to orbital mixing and the presence of d orbital vacancies, even in Au^0^ or Au^1+^ states. This can permit the occurrence of the 2p_3/2_ → 5d transition in which the intensity of this peak appears to be strongly dependent on the corresponding unoccupied d states, which strongly depends on the electronic charge transfer between the absorbing atoms and ligands.^28^

**Figure 1:**
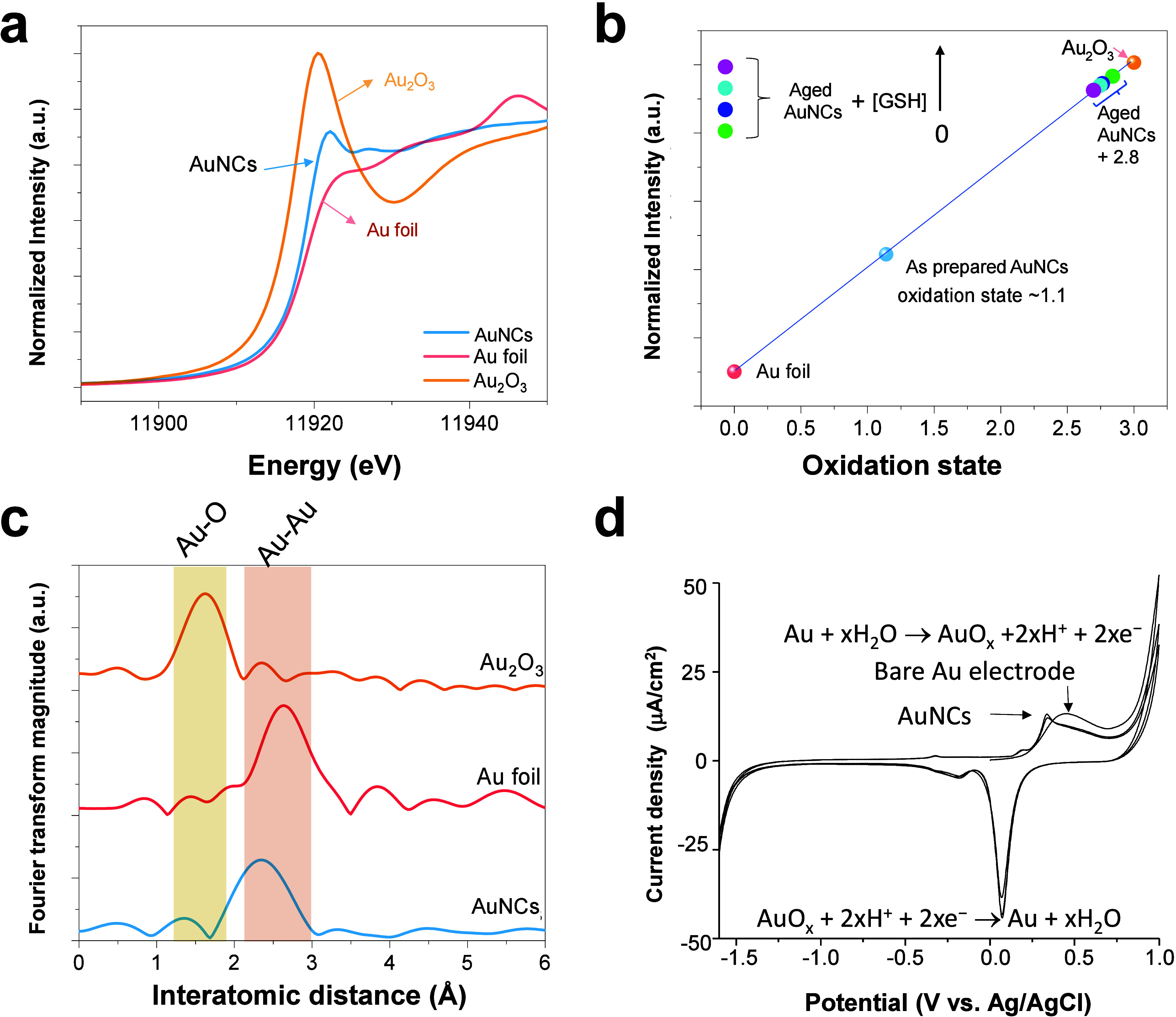
Gold nanoclusters (AuNCs) containing Au–O species form an electron-deficient state. (a) XANES spectra of the Au L_3_-edge of AuNCs and references (Au foil and Au_2_O_3_). (b) A linear plot of the oxidation states of fresh and aged AuNCs and two references were created on the basis of the normalization intensity of XANES. (c) Corresponding Fourier transform–EXAFS spectra of AuNCs and the two references. (d) Redox potential of AuNCs by cyclic voltammograms of AuNCs in 0.1 N NaOH with a scan rate of 0.1 V s^–1^. Bulk gold as a working electrode and the electrolyte solution (pH 8.5–9.0) containing AuNCs (0.1 mM) and bubbling O_2_ for 10 min before scanning.

It should be noted that the XANES spectrum of AuNCs in the present study was characteristic of a considerably larger white line intensity than that of the Au(0) bulk. The increase in the intensity of the white line (Figure 1a), as mentioned earlier, can be attributed to the fact that the unoccupied d states of AuNCs were considerably larger than those of Au^0^. The further quantification of the unoccupied d states (i.e., oxidation state) through a calibration curve based on the white line intensity of references, as plotted in Figure 1b, clearly showed that the average oxidation state of fresh AuNCs (as-prepared) was about +1.1, indicating that the as-prepared AuNCs possessed an elevated oxidation state, as well as unusual frontier orbitals, compared with conventional gold nanoparticles. Moreover, the aged AuNCs could increase the oxidation state to about +2.8, which was not affected in the coexistence of high-concentration glutathione. This phenomenon may be attributed to the superficial gold atoms enriching the oxygen intercalation sites to form Au–O bonding, which is further supported by the finding from Fourier transform spectra of the extended X-ray-absorption fine-structure (EXAFS), as illustrated in Figures 1c and S1, and indicates the formation of electron-deficient AuNCs. Notably, Figure 1d shows the oxidation potential of AuNCs appearing at +0.334 V (vs Ag/AgCl) that is evidently lower than that of bulk bare gold electrode. This phenomenon can be attributed to the Plieth equation,^29,30^ which states the ultrasmall size gold nanoparticles (i.e., AuNCs) can undergo oxidation at an unusually low potential. The result confirms that the AuNCs could facilely form Au^δ+^ clusters, a state with a high electron affinity for accepting electron. Such a unique characteristic might facilitate a CoQ-like electron-demanding state as a RET trapper (vide infra) to capture mitochondrial reverse electrons.

In addition, the EXAFS spectrum that originates from the interference features induced by backscattering photoelectrons of neighboring atoms can accordingly explain the local coordination environment around the absorbing atoms (i.e., coordinated element, coordination number, and interatomic distance). The Fourier transform EXAFS spectra of AuNCs L_3_-edge exhibited one distinctive peak at around 2.4 Å for the Au–Au scattering path (Figures 1c and S1), but characteristic peaks of metallic second-shell Au–Au bonds around 3.5–4.1 Å in the AuNCs were nonexistent, which clearly indicated poor crystalline nature or nanocluster existence without long-range order. To further consider the atomic configuration of AuNCs, which apparently refers to the nature of its electron transfer with capping molecules,^31,32^ a standard fitting procedure quantifying the local environment of Au atoms was performed. The extracted interatomic distance of the Au–Au scattering path was determined to be approximately 2.51 Å (Figure 2a), revealing a typically regular pattern (Figure 2b). It is noteworthy that in addition to the metallic Au–Au scattering path, a remarkably intensive contribution at approximately 1.5 Å in the EXAFS spectrum was attributed to the Au–O scattering path. This finding clarifies the fact that these surface low-coordinated Au atoms were stabilized by forming Au–O bonds, which may be caused by capping agents during the synthesis and corroborate the XANES results. This observation further supports the phenomenon of the extracted interatomic distance of the Au–Au path (i.e., ∼2.5 Å) in the cluster sample being considerably shorter than that of bulk Au (2.88 Å), which corroborates our XANES result illustrated in Figure 1c. Notably, the nature of AuNCs is strongly dependent on the corresponding atomic configuration, which can be further validated by quantitative analysis of the coordination number (CN) for AuNCs. The inset table in Figure 2 shows that a CN of 3.8 (4) was clarified. Such a CN value can be attributed to the formation of a Cs symmetry that refers to a theoretical CN of 3.875 (Figure 2c) for such an Au_8_ cluster, as demonstrated by the mass spectrum (Figure S2) analyzed in our previous study.^33^ These key features provide chemical molecular insights into AuNCs, which may potentially be developed as CoQ-like mimics of an electron acceptor (i.e., RET trapper) in abnormal mitochondria.

**Figure 2:**
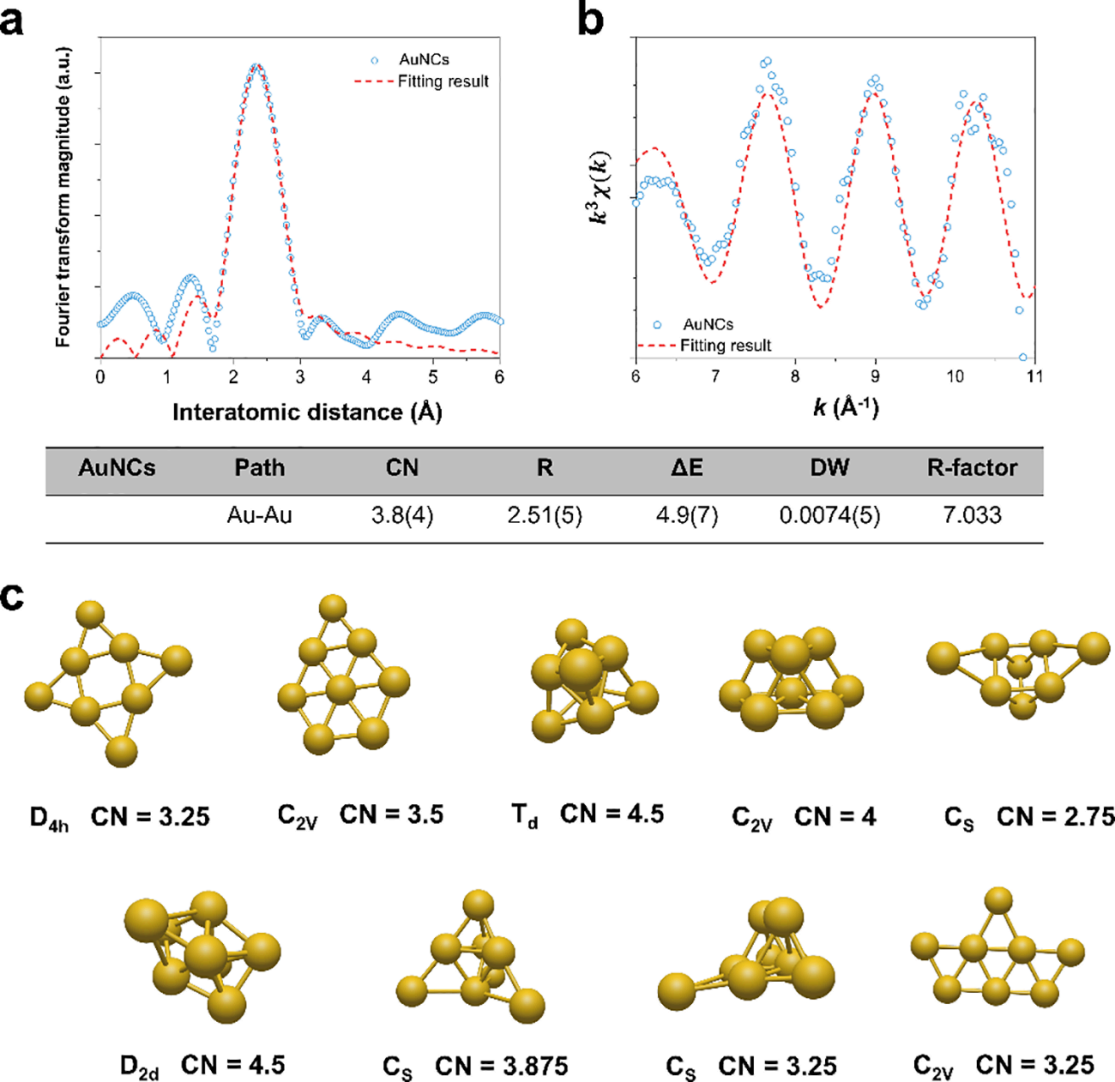
EXAFS fitting curves for gold nanoclusters (AuNCs) at the R-space (a) and K-space (b). The inset table provides structural parameters of AuNCs extracted from EXAFS fitting. (c) Atomic configuration of the eight-atom Au cluster (Au_8_) and corresponding theoretical coordination number (CN). Abbreviation: *R*, interatomic distance; Δ*E*, delta *E*; DW, Debye–Waller factors.

For *in vitro* models, we used 3D-cultured spheroidal HepG2 cells that have been reported to show liver-like phenotypes to determine whether AuNCs possess CoQ-like activity.^34^Figure S3 shows specific characteristics of liver-like phenotypes in the spheroidal HepG2 cells of our building culture system. Additionally, the confocal images show colocalization of AuNCs and mitochondria labeled with MitoTracker dye in HepG2 cells (Figure S4), which indicates the appearance of AuNCs at the mitochondria. It should be emphasized AuNCs do not show superoxide dismutase (SOD)-like activity,^35^ so the role of scavengers in decreasing the level of mitochondrial superoxide anions can be excluded. To demonstrate the possible efficacy of AuNCs acting as a RET trapper, we used oligomycin and 2-deoxyglucose (2DG) to mimic the IRI condition with transient ischemia by inhibiting ATP synthesis and glucose uptake, respectively.^36,37^ The conceptual illustration is plotted in Figure 3a. As expected, confocal microscopic imaging showed that the spheroidal HepG2 cells treated with oligomycin and 2DG to elicit a metabolic shift toward IRI had the highest mitochondrial fluorescence intensity (Figure 3b,c), indicating a high level of mitochondrial superoxide anions. Notably, the measurement of mitochondrial fluorescence intensity originated from a specific mitochondrial superoxide detection probe (i.e., MitoSOX). Comparatively, the spheroidal HepG2 cells with the IRI phenotype treated with AuNCs showed significantly lower fluorescence intensity (Figure 3b,c). The results strongly suggested that the AuNCs could efficiently capture the electrons leaked during RET by acting as a CoQ. To further confirm this result, the spheroidal HepG2 cells were incubated under low oxygen to mimic hypoxic conditions before intentional recovery to normoxia, which mimicked the cell-based ischemia-reperfusion process. The results of this experiment also showed that the MitoSOX fluorescence signal of spheroidal HepG2 cells was reduced in the presence of AuNCs (Figure 3d). These findings indicated that the CoQ-like AuNCs might contribute to avoiding the formation of RET-dependent mitochondrial superoxide anions. More importantly, the unique CoQ-like activity could not be observed when using AuNCs more than 2 nm and less than 20 nm in size (Figure S5).

**Figure 3:**
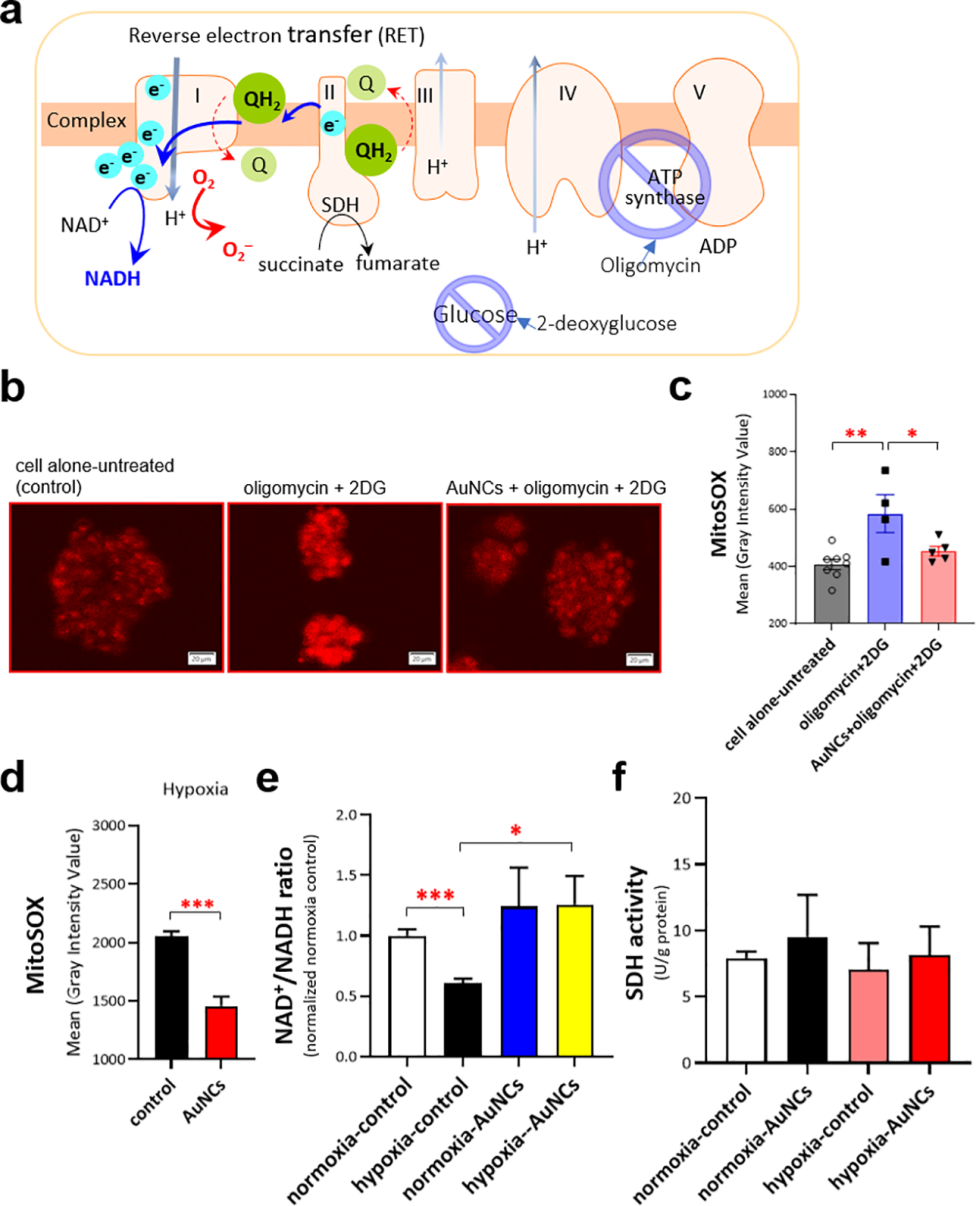
Gold nanoclusters (AuNCs) could be CoQ-like mimics to receive reversed electrons in an ischemia-reperfusion injury (IRI)-mimicking *in vitro* model. (a) A conceptive plot shows how to produce the IRI-mimicking *in vitro* model. (b) Representative confocal microscopic imaging shows the MitoSOX fluorescence intensity of spheroidal HepG2 cells treated with oligomycin, 2DG, and AuNCs for 30 min. (c) The mean fluorescence intensity of spheroidic HepG2 cells from (b) was quantified by Olympus cellSens dimension desktop software. *P* values were obtained by one-way ANOVA with Tukey’s multiple comparisons test. (d) The mean fluorescence intensity of spheroidal HepG2 cells under low oxygen pressure (1% O_2_, hypoxic condition) for 30 min was quantified in the presence of AuNCs (*n* = >30 spheroidic HepG2 cells per group). Unpaired, two-tailed Student’s *t* tests were performed to obtain *p* values. (e) AuNCs contributed to the restoration of the NAD^+^/NADH ratio. The data represent an average of three independent experiments, and unpaired, two-tailed Student’s *t* tests were performed to obtain the *P* value. (f) AuNCs uptake could not enhance SDH activity. Data represent an average of four independent experiments. (*, *P* < 0.05; **, *P* < 0.01; ***, *P* < 0.001)

It should be noted that once respiratory complex I receives reversed electrons, NAD^+^ is reduced to NADH, leading to a lower NAD^+^/NADH ratio (Figure 3e, black versus white bars). In contrast, under hypoxia-mimicking conditions, AuNCs captured a few back-transferred electrons and significantly increased the NAD^+^/NADH ratio, similar to the normoxic spheroidal HepG2 cells treated with AuNCs (Figure 3e, yellow versus blue bars), thereby indicating that AuNCs retarded hypoxia-induced RET to complex I. This result might also reflect the high electron capture tendency of AuNCs that can act as a CoQ–nanozyme to relax the RET. Accordingly, SDH in mammalian mitochondria shows a diodelike property that controls the forward electron flow to the CoQ pool in one direction only.^38^ Therefore, we also assessed SDH activities during RET in the presence of CoQ-mimicking AuNCs, which might compensate for the consequences of the CoQ–redox pool cycle and RET. However, no contribution to SDH activity was observed under either normoxia or hypoxia in the presence of AuNCs (Figure 3f). Our results suggest that AuNCs may optimize the balance of mitochondrial electron supply and demand during IRI-induced RET.

To further evaluate whether AuNCs can serve as a potential electron acceptor in protective therapeutic strategies for targeting RET and confirm the abovementioned in vitro model results, we chose the hepatic IRI mouse model (Figure 4a, left panel). As expected, a similar feature in the liver accumulation of nanoparticles has been found in our AuNCs in the biodistribution of the hepatic IRI model (Figure 4a, right panel). Meanwhile, the kidney accumulation for renal excretion was also shown (urine data not shown). After treatment, hepatic histopathology showed an overall reduction in the injury area in AuNC-treated mice with ischemia-reperfusion (Figure 4b), whereas severe pathological changes in the liver were observed in the mice progressing to IRI. These data were graded using Suzuki’s histological scores (Figure 4c).^39^ After IRI, damaged cells release high-mobility group box 1 (HMGB1) protein, a damage-associated molecular pattern (DMAP) molecule.^40^ Immunohistochemical analysis showed a high expression of HMGB1 within the cytoplasm of hepatic tissues (area in brown color) in mice with IRI, while HMGB1 was expressed in the nuclei (brown circles) of hepatocytes in AuNC-treated mice (Figure 4d). Quantitative analysis of HMGB1 in liver tissue showed that HMGB1 expression increased in liver tissue of mice progressing to IRI; in contrast, treatment with AuNCs inhibited these effects (Figure 4e). It is well known that HMGB1 in liver IRI has a paracrine effect and is an early mediator of inflammation.^41^ The downstream inflammatory marker, pNF-kB, was quantified by using immunohistochemistry (Figure 4f). The serum IL-6 level, which is correlated to systemic inflammation, decreased slightly in the AuNC-treated mice (Figure 4g). The serum biomarkers associated with liver function, including GOT, GPT, and LDH, were also rescued from IRI by treatment with AuNCs (Figure 4h).

**Figure 4:**
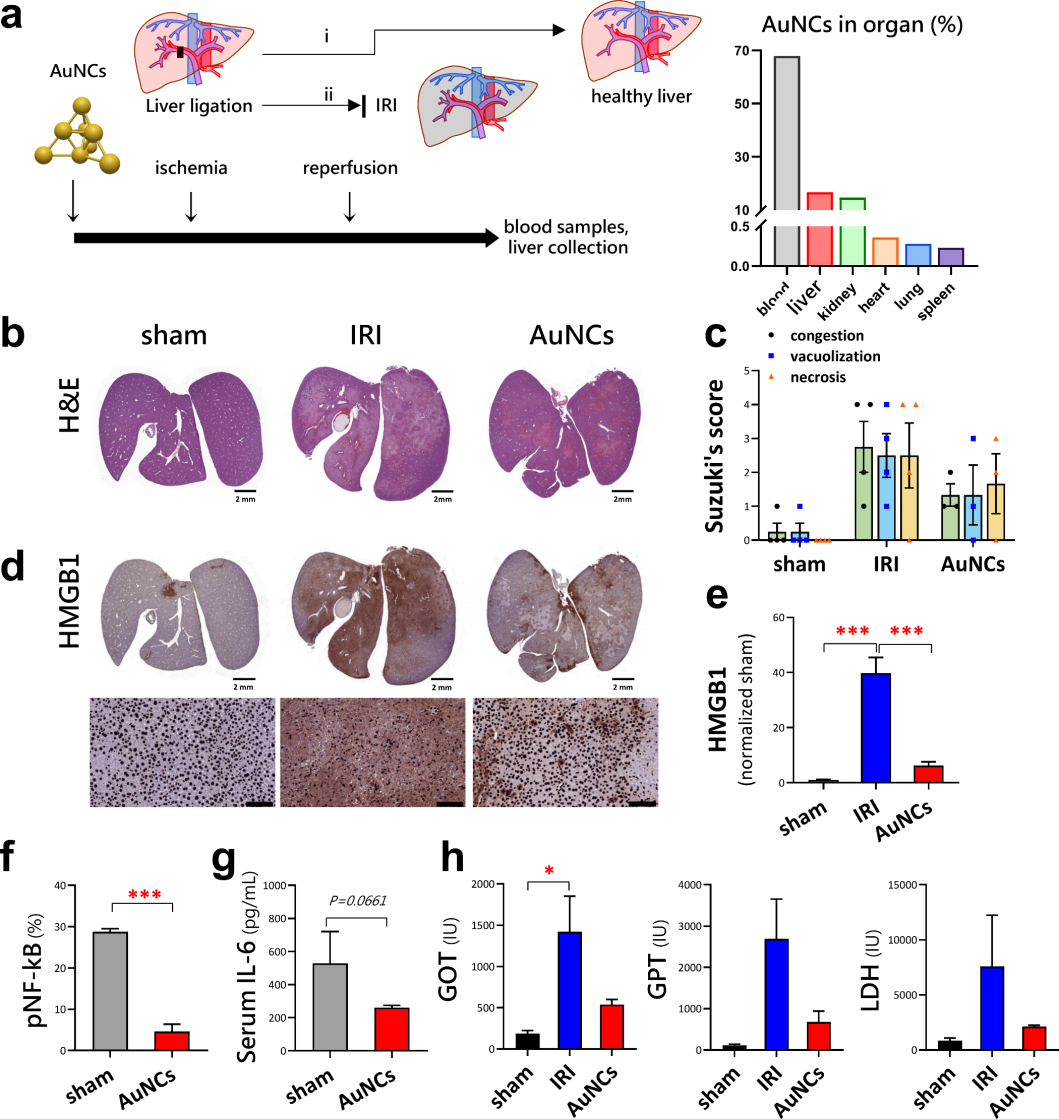
Gold nanoclusters (AuNCs) protected mouse livers against ischemia-reperfusion injury (IRI). (a) Model of hepatic IRI in mice (i) with or (ii) without AuNC precondition treatment. The right panel shows the biodistribution of AuNCs after a two-hour time point of injection. The liver illustration was recreated using illustration toolkits purchased from Motifolio. (b) Hematoxylin and eosin (H&E) staining of all hepatic sections with IRI. Scale bar: 2 mm. (c) Suzuki’s score was used to quantify the severity of congestion, vacuolization, and necrosis of hepatocytes in H&E-stained liver sections from (b). (d) The upper panel shows an immunohistochemical (IHC) analysis of HMGB1 protein expression of all liver sections. Scale bar: 2 mm. The bottom panel shows a magnification of IHC analysis of HMGB1. Scale bar: 100 μm. (e) Quantification of the IHC image for HMGB1 in liver sections from (d). (f) Quantification of IHC analysis of pNF-kB protein expression of liver sections with IRI. (g,h) Serum IL-6, GOT, GPT, and LDH concentrations of mice. (*, *P* < 0.05; ***, *P* < 0.001)

In summary, our study shows that unique AuNCs with a low-coordination configuration are capable of performing an electron-deficient CoQ-like function for capturing mitochondrial reverse electrons and consequently preventing IRI to organs. The AuNCs were composed of eight atoms (Au_8_) forming a Cs-symmetrical configuration that allowed all gold atoms to be exposed on the surface to enhance the likelihood of oxygen intercalation. Therefore, the AuNCs showed an unusually high oxidation state compared to conventional gold nanoparticles and attained an electron-demanding state similar to that of CoQ, which enabled the prompt capture of electrons involved in mitochondrial RET and subsequent inhibition of mitochondrial superoxide anion formation. Moreover, using hepatic IRI cell/animal models, we corroborated the AuNCs’s CoQ-like activity and their innovative therapeutic role in avoiding inflammatory adaptation and preventing liver dysfunction caused by IRI.

## Supporting Information Available

Materials and methods, including the details of sample preparation and characterization of X-ray absorption spectra measurement and fitting equation of the Fourier transform-EXAFS spectra for low-coordinated gold nanoclusters (AuNCs), 3D-spheroid HepG2 cells and liver ischemia-reperfusion injury (IRI) for in vitro and in vivo models, serum biochemical measurements, mitochondrial MitoSOX Red staining, PCR quantification, histology and immunohistochemistry, and other associated Tables and Figures (PDF)
