# Supplementary material for: Oxygen-Binding Sites of Enriched Gold Nanoclusters for Capturing Mitochondrial Reverse Electrons
Source: Nano Lett. 2024 Aug 29;24(36):11202–9. doi: 10.1021/acs.nanolett.4c02331 (PMC11403762; doi:10.1021/acs.nanolett.4c02331)
Supplement: Supplementary file 1 — nl4c02331_si_001.pdf [file nl4c02331_si_001.pdf]

Supporting Information

Oxygen-binding sites of enriched gold nanoclusters for capturing mitochondrial reverse electrons

Fang-Hsuean Liao,[†] Shu-Ping Chen,[†] Chun-Nien Yao,[†] Te-Haw Wu,[†] Meng-Ting Liu,[‡] Chia-Shuo Hsu,[‡] Hao Ming Chen,[‡,§,] and Shu-Yi Lin[†,‖,]**

[†] *Institute of Biomedical Engineering and Nanomedicine, National Health Research Institutes, Zhunan Town 35053, Taiwan*

[‡] *Department of Chemistry and Center for Emerging Materials and Advanced Devices, National Taiwan University, Taipei 106319, Taiwan*

[§] *National Synchrotron Radiation Research Center, Hsinchu300092, Taiwan*

[‖] *Department of Chemistry, National Tsing-Hua University, Hsinchu 300044, Taiwan*

MATERIALS AND METHODS

1. Synthesis of AuNCs
2. Cell culture and experiments
3. Quantification PCR
4. Animals
5. Serum biochemical measurements
6. Histology and immunohistochemistry
7. Statistical analyses
8. X-ray absorption spectra (XAS) measurement
9. EXAFS fittings
10. Table S1
11. Figure S1-S4

12. reference lists

MATERIALS AND METHODS

Synthesis of gold nanoclusters (AuNCs)

The amine or hydroxy-terminated and low-generation dendrimers and other dendrimers and $HAuCl_4$ were purchased from Sigma, Inc. (San Diego, CA, USA); an MWCO membrane filter was purchased from Millipore (PES membrane); and an anion exchange resin was purchased from (Merck, Fractogel® EMD TMAE Hicap). The ultra-small nano-gold (AuNCs) were synthesized using a previously published method (number 1 listed in Table S1).[1] First, varying amounts of $HAuCl_4$ or $HAuBr_4$ (Sigma-Aldrich, 400 μL, 60 μmol, 150 mM) were added into 20 mL of deionized water containing the low-generation dendrimers (Aldrich, 874 μL, 46 μmol, 10 wt % methanol solution). Each dendrimer and $HAuCl_4$ mixed solution was then incubated at 4°C overnight before being irradiated with microwaves (CEM, Discover LabMate System, 300W/120°C for 30 min). After reduction, the precipitates and AuNCs were filtered through a 3 KDa MWCO PES membrane filter (Millipore, Amicon Ultra), and the extra anion, such as $AuCl_4^-$, was removed by an anionic exchange chromatograph to obtain the purified AuNCs precursor from G_4NH_2. The G_2OH dendrimer to prepare the AuNCs for mass measurement can be much more accessible to measure, with the results displayed in Figure S2. To do cyclic voltammograms, the AuNCs solution (0.1 mM, 10 mL) was adjusted to pH 8.5–9.0 by dropwise addition of NaOH solution (50 mM). The mixture was saturated by bubbling O_2 for 10 min before scanning.

Cell culture and experiments

HepG2 cells were incubated in MEM medium supplemented with 10% (v/v) heat-inactivated FBS, 1 mM sodium pyruvate, and non-essential amino acids at 37 ˚C under a humidified normoxic atmosphere with 5% CO_2. HepG2 cells were plated at 80,000 cells per well in 96-well Tantti SpherTantrix 3D dissolvable scaffold plates (Tantti® Laboratory Inc., Taiwan) to form three-dimensional (3D) spheroids. After four days, 3D-spheroid HepG2 cells were suspended using SpherTantrix dissolving solution (Tantti® Laboratory Inc., Taiwan) according to the manufacturer's protocol and then plated in ultra-low-attachment culture dishes (Alpha Plus Scientific Corp., Taiwan) until processing for experiments. On day 5, the 3D-spheroid HepG2 cells were treated with 12.5 ug/mL AuNCs in mitochondrial assay solution (MAS) buffer for 30 min under hypoxic conditions to mimic oxygen and nutrient deprivation and harvested for the following experiments: mitochondria Mito-Tracker Green (Molecular Probe Inc., OR, USA) staining; mitochondrial MitoSOX Red staining (Molecular Probe Inc., OR, USA); NAD/NADH colorimetric assay (Abcam, Cambridge, MA, USA); intracellular succinate dehydrogenase activity (Elabscience Biotechnology Inc., Texas, USA); and liver-like phenotype gene expression analysis using RT-PCR.[2] We defined hypoxic conditions as 1% O_2 in a cell culture incubator. The MAS buffer (pH 7.2) consisted of 70 mM sucrose, 220 mM mannitol, 10 mM KH_2PO_4, 5 mM $MgCl_2$, 2 mM HEPES, 1 mM EGTA, and 0.2% (w/v) BSA.

HepG2 cells were treated with 12.5 μg/mL AuNCs for 30 min and stained with MitoTracker Green (Invitrogen), a cell-permanent probe for labeling mitochondria, for colocalization analysis of AuNCs (ZEISS, LSM 900, Germany). Cells were treated with 12.5 μg/mL AuNCs for 30 min under normoxic or hypoxic conditions, and then stained with 5 μM MitoSOX Red (Molecular Probe Inc.) for 10 min at 37 °C while protected from light. MitoSOX Red superoxide indicator has maximum excitation and emission at 510 nm and 580 nm, respectively. The stained cells were transferred into a μ-slide 8-well plate (catalog number 80826, ibidi, Munich, Germany) to facilitate image capture. Images of stained cells were captured using a FV10i confocal microscope (Olympus). Then, the MitoSOX fluorescence intensity of spheroidal HepG2 cells (estimated cell number from 10^5~10^6 per spheroidic cell) with a lateral area of more than 100 μm^2 was quantified by Olympus cellSens dimension desktop software.

PCR quantification

To validate liver-like phenotype gene expression of spheroid HepG2, RNA was extracted using the Direct-zol™ RNA MiniPrep kit (Zymo Research, Irvine, CA, USA) and quantified using a BioTek Synergy H1 microplate reader (BioTek® Instruments, Inc., VT, USA). One microgram of RNA was reverse transcribed into cDNA using the Maxima First Strand cDNA Synthesis kit (Thermo Fisher Scientific Inc., Waltham, MA, USA) with a PCR machine

(Mastercycler X50s; Eppendorf, Hamburg, Germany). For gene transcription, cDNA samples were analyzed using the KAPA SYBR® FAST qPCR master mix kit (KAPA Biosystems, Cape Town, South Africa), with 300 nM primers specific to the genes of interest, and Real-Time PCR AB QuantStudio™ 5 with QuantStudio™ Design & Analysis Software (Thermo Fisher Scientific Inc.). Relative expression was normalized to the housekeeping gene beta-actin and calculated using $2^{-\Delta\Delta Ct}$ methods.

Animals

All procedures were performed according to the guidelines approved by the Institutional Animal Care and Use Committee (IACUC, NHRI-IACUC-108135A), NHRI. C57BL/6 wild-type male mice, 8–12 weeks of age, underwent surgery to cause liver ischemia-reperfusion injury (IRI).[3] Briefly, all mice were injected intraperitoneally with the analgesic buprenorphine (2 mg/kg) and randomly divided into three groups: the sham group and two IRI model groups pretreated with an intravenous injection of either AuNCs (10 mg/kg) or PBS 30 minutes before the surgery. Mice were anesthetized by inhalation of 2% isoflurane with an isoflurane anesthesia system and placed on a heating pad to maintain body temperature. The sham group underwent the same surgical procedure without arterial clip clamping. The two IRI groups were subjected to 30 minutes of 70% liver ischemia by cross-clamping the hepatic artery and portal vein to the left and median hepatic lobes using an

atraumatic clip to deprive blood flow. After six hours of reperfusion, mice were euthanized with a high dose of isoflurane, their blood was collected by cardiac puncture, and liver tissue was collected for biochemical measurements and histological staining analysis. To measure AuNCs biodistribution, mice were injected intraperitoneally with AuNCs. After two hours, organs were removed and weighed, and Au-atom content was measured using inductively coupled plasma mass spectrometry (ICP-MS). We quantified the Au-atom content for each organ and divided it by the injected Au-atom content per mouse to calculate AuNCs in organs (%).

Serum biochemical measurements

The levels of serum glutamate oxaloacetate transaminase (GOT), glutamate pyruvate transaminase (GPT), and lactic dehydrogenase (LDH) are specific to liver cell injuries and were measured using a fully automatic biochemical analyzer. For cytokine analysis, the concentrations of serum IL-6 were determined using the LEGEND MAX™ mouse IL-6 ELISA kit (Biolegend, Inc., San Diego, CA, USA).

Histology and immunohistochemistry

After euthanasia, the left and median lobes of the liver of each mouse were quickly removed and fixed in ice-cold 10% phosphate-buffered formalin for 24 h. The experimental

procedures for histology (including paraffin-embedding and sectioning) and immunohistochemistry were performed by the Pathology Core Laboratory of NHRI. Liver paraffin sections were stained with hematoxylin and eosin (H&E) and immunostained with the primary antibody of HMGB1 diluted at 1:100 (Cat#ab79823, Abacm) and phospho-Ser536 NF-κB p65 diluted at 1:20 (Cat#3033S, Cell Signaling Technology, Inc., Danvers, MA, USA). To evaluate liver injury, H&E-stained liver sections were obtained from four different regions and graded using Suzuki's histological score from 0 to 4 according to the severity of congestion, vacuolization, and necrosis of hepatocytes. A score of 0 meant no pathological change, whereas a score of 4 indicated severe congestion and more than 60% lobular necrosis. Quantification of HMGB1 and phospho-Ser536 NF-κB from five different regions of each liver section was performed using ImageJ.

Statistical analyses

We used GraphPad Prism (v9.5.1) to input data. Data are expressed as mean ± SEM. Statistical differences were determined using one-way ANOVA with Tukey's multiple comparisons test and unpaired, two-tailed Student's *t*-tests to determine statistical significance (*, $P<0.05$; **, $P<0.01$; ***, $P<0.001$).

X-ray absorption spectra measurement

X-ray absorption spectroscopy (XAS) data were obtained in total-fluorescence mode at BL12B2 of Taiwan beamline at Spring-8 of National Synchrotron Radiation Research Center (NSRRC). The electron storage ring ran at 8.0 GeV with a steady current of approximately 400 mA. The incident beam energy was refined using a Si (111) double-crystal monochromator. The scan range was maintained within an energy span of 11720–11716 eV for the Au L_3-edge. The data collected underwent normalization based on the incoming incident photon flux and were processed using Athena software from the IFEFFIT package. To calibrate all the data, E_0 values of 11919 eV were employed, referencing the first inflection point of the gold foil absorption L_3-edge. Fourier transform analysis of k^3-weighted extended X-ray absorption fine structure (EXAFS) oscillations was then performed to assess the contribution of each bond pair to the Fourier transform peak during the EXAFS analysis.

EXAFS curve fitting

EXAFS curve fitting was performed with REX2000 software using ab initio-calculated phases and amplitudes from the FEFF 8.2 program. These ab initio phases and amplitudes were used in the EXAFS equation:

$$\chi(\kappa) = \sum_i \frac{N_i F_i(k)}{kR_i^2} \cdot S_0^2 \cdot exp^{(-2k^2\sigma_i^2)} \cdot exp^{(^{-2R_i}/_{\lambda})} \cdot sin^{(2kR_i+\delta_i(k))}$$

The distances from the neighboring atoms to the central atoms are divided into *i* shells, with all atoms with the same atomic number and distance from the central atom grouped into a

single shell. Within each shell, the coordination number N_i denotes the number of neighboring atoms in shell i at a distance of R_i from the central atom. The ab initio amplitude function for shell i and the Debye–Waller term $e^{-2\sigma i2k2}$ accounts for damping from static and thermal disorder in absorber–backscattered distances. S_0^2 is an amplitude reduction factor due to shake-up/shake-off processes at the central atom(s). The EXAFS equation was used to fit the experimental data using N, R, and the EXAFS Debye–Waller factor (DW; σ^2) as parameters. For the energy (eV) to wave vector (k, Å^{-1}) axis conversion, the S_0^2 value was determined as 0.90.

Table S1. Listed our previous publications for the novelty of polymer-templated gold nanoclusters (AuNCs)

polymers	Encapsulation	novelty	Reference literatures
PAMAM dendrimers	AuNCs	A synthetic approach for AuNCs with high quantum yield	1. *Chem. Commun.* **2010**, 46, 2626–2628.
PAMAM dendrimers		Structural breakdown of PAMAP dendrimers induce the intricate luminescence	2. *Chem. Eur. J.* **2013,** 19, 11672–11675.
PAMAM dendrimers	AuNCs	Deformation, collapse, and detoxification of various generations of PAMAP dendrimers induced by the AuNCs can increase PAMAM biocompatibility and change their intracellular trafficking	3. *J. Mater. Chem.*B **2014**, 2, 6730–6737.
PAMAM dendrimers	AuNCs	Surface polarity changes the self-assembly polymorph of biomolecule	4. *Nano Lett.* **2015**, 15, 6446–6453.
PAMAM dendrimers	AuNCs	Confined the packing density of bio-supermolecules inhibit toxicity immunological responses application and showed body excretion of AuNCs	5. *Nano Lett.* ***2018,*** *18, 2864-2869.*
PAMAM dendrimers	AuNCs	Elucidated how the atomic configuration of AuNCs in response to their inherent antioxidant competence	6. The work

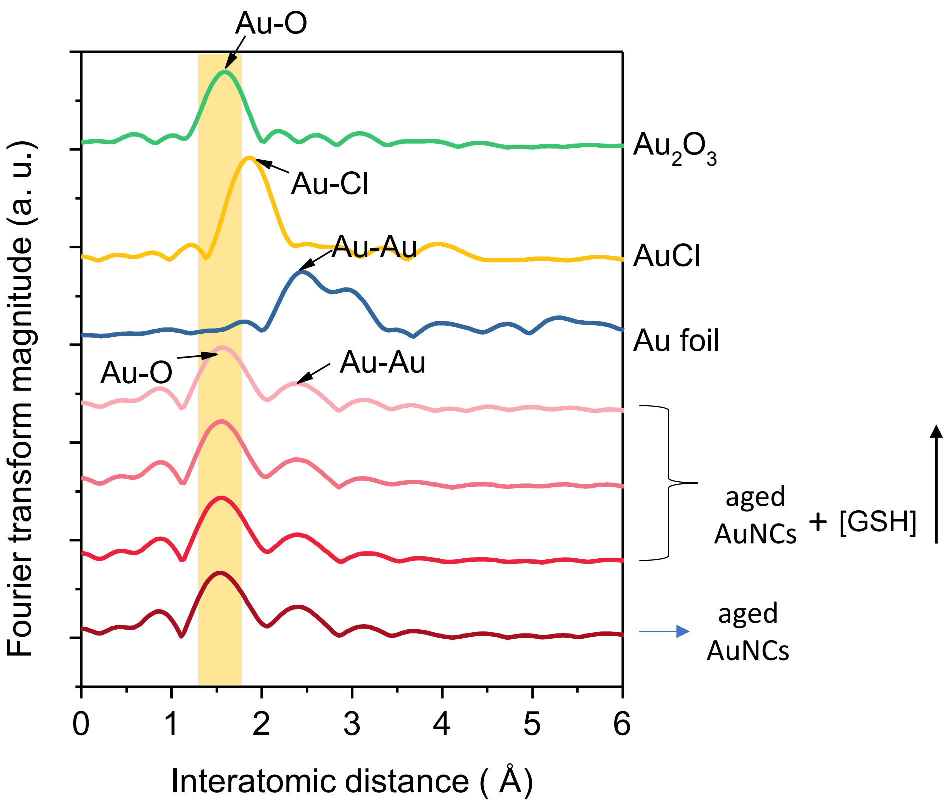

Figure S1. Aged gold nanoclusters (AuNCs) dramatically increase Au-O species forms in an electron-deficient state. Corresponding Fourier transform (FT)-EXAFS spectra of AuNCs and control references (Au foil, AuCl, and Au_2O_3).

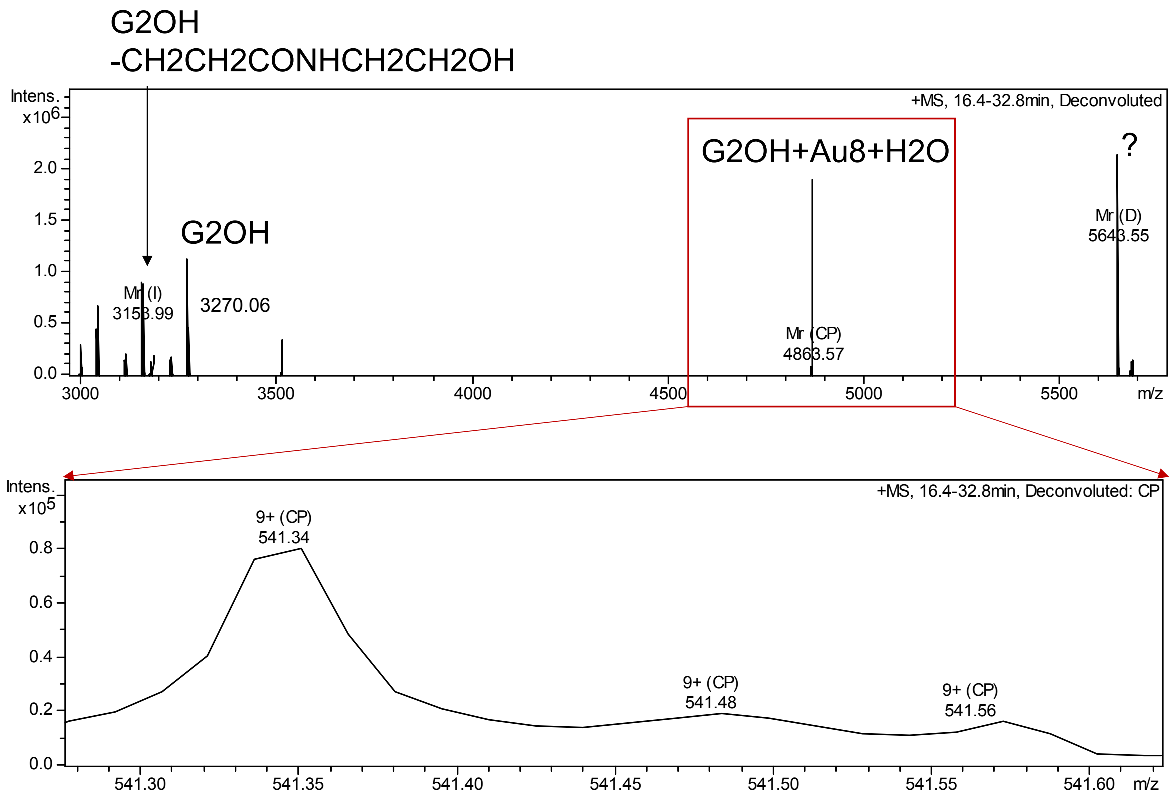

Figure S2. ESI-mass spectra of G_2OH-encapsulated gold nanoclusters (AuNCs, Au_8).

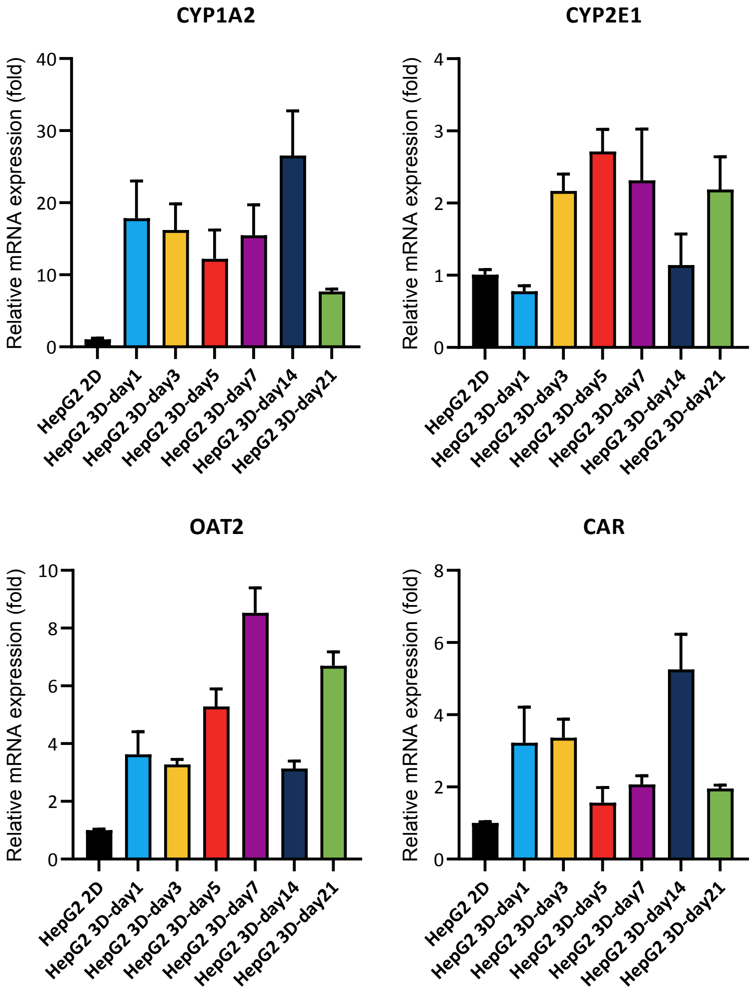

Figure S3 Spheroidal 3D-cultured HepG2 cells exhibit liver-like phenotypes, i.e., specific drug-metabolizing enzymes. The mRNA levels of phase I xenobiotic metabolizing enzymes (CYP1A2 and CYP2E1) and representative major transporters of drugs (organic anion transporter 2, abbrev: OAT2), as well as of nuclear receptors (constitutive active/androstane receptor, CAR gene), were validated and increased in 3D culture compared to 2D culture.

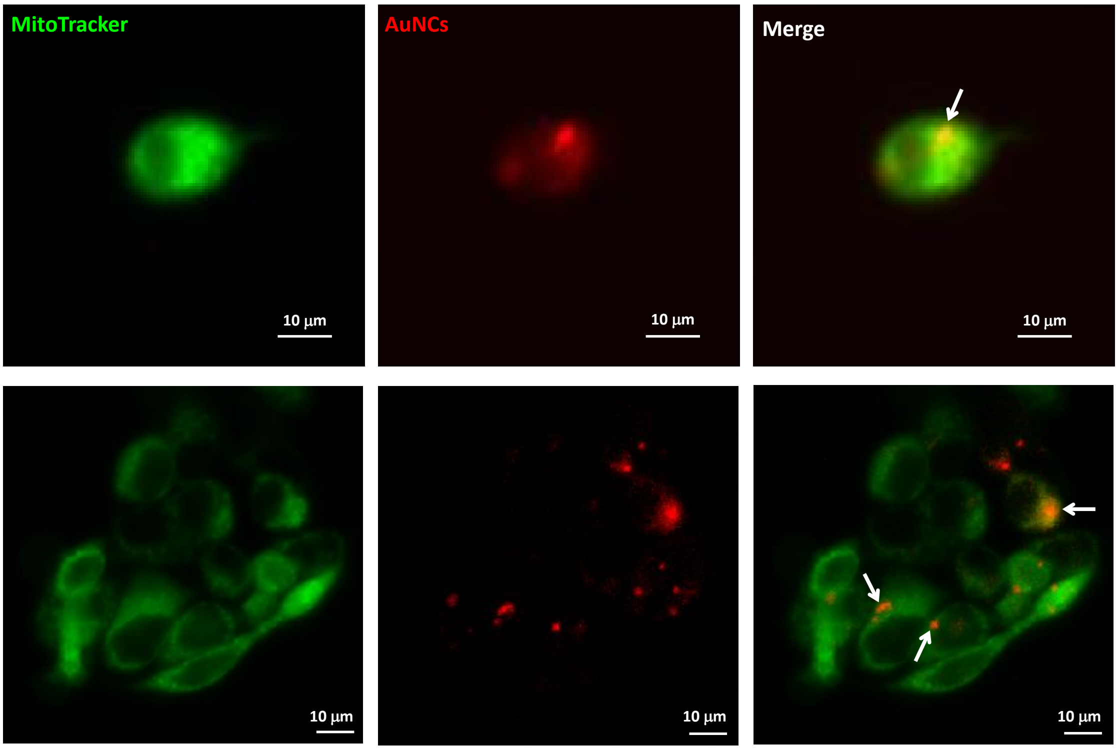

Figure S4. Confocal microscopy images display yellow and orange dots (white arrows) indicating the colocalization of AuNCs (shown in red) with mitochondria labeled with MitoTracker Green in HepG2 cells. In these images, we represent the blue fluorescence of AuNCs with red to avoid ambiguity due to the overlap with green.

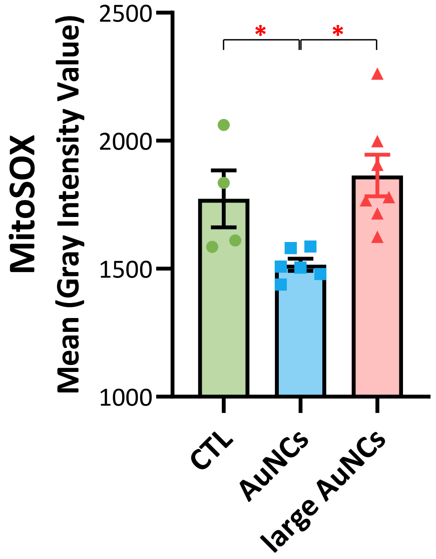

Figure S5. The fluorescence intensity of MitoSOX in spheroidal HepG2 cells treated with gold nanoclusters (AuNCs) of different sizes. These results indicate that AuNCs might contribute to the avoidance of RET-dependent superoxide anion formation, but this result disappears when using AuNCs more than 2 nm and less than 20 nm in size (large AuNCs).

Reference lists:

(1) Jao, Y. C.; Chen, M. K.; Lin, S. Y. Enhanced quantum yield of dendrimer-entrapped gold nanodots by a specific ion-pair association and microwave irradiation for bioimaging. *Chem. Commun.* **2010**, *46* (15), 2626-2628.

(2) Ramaiahgari, S. C.; den Braver, M. W.; Herpers, B.; Terpstra, V.; Commandeur, J. N.; van de Water, B.; Price, L. S. A 3D in vitro model of differentiated HepG2 cell spheroids with improved liver-like properties for repeated dose high-throughput toxicity studies. *Arch. Toxicol.* **2014**, *88* (5), 1083-1095.

(3) Abe, Y.; Hines, I. N.; Zibari, G.; Pavlick, K.; Gray, L.; Kitagawa, Y.; Grisham, M. B. Mouse model of liver ischemia and reperfusion injury: method for studying reactive oxygen and nitrogen metabolites in vivo. *Free Radic. Biol. Med.* **2009**, *46* (1), 1-7.
